# Recommendations for the diagnosis and management of cln3 disease (batten disease) using the Delphi consensus methodology

**DOI:** 10.1186/s13023-026-04298-2

**Published:** 2026-03-10

**Authors:** Jonathan W. Mink, Heather R. Adams, Rebecca Ahrens-Nicklas, Brian Nauheimer Andersen, Erika Augustine, Rose‑Mary Boustany, Jonathan D. Cooper, Alex Levin, Paul Gissen, Minna Laine, Heather L. Mason, Sara E. Mole, Miriam Nickel, John R. Ostergaard, Lori Sikorra, Lauren Treat, Ineka T. Whiteman, Ruth Williams, Angela Schulz

**Affiliations:** 1 Pittsford, NY USA; 2 University of Rochester Medical Center, Rochester, NY USA; 3 The Children’s Hospital of Philadelphia, University of Pennsylvania, Philadelphia, PA USA; 4 Centre for Rare Diseases, Aarhus University Hospital, Aarhus, Denmark; 5 Kennedy Krieger Institute, Baltimore, MD USA; 6 American University of Beirut, Beirut, Lebanon; 7 Washington University in St Louis, School of Medicine, St. Louis, MO USA; 8 Flaum Eye Institute, University of Rochester, Rochester, NY USA; 9 UCL Great Ormond Street Institute of Child Health, University College London, London, UK; 10 Helsinki University Hospital, Helsinki, Finland; 11 Coufetery Comms, Medical Writing Services, Paris,, France; 12 Children’s Hospital, University Medical Center Hamburg-Eppendorf, Hamburg, Germany; 13 Los Robles Hospital and Medical Center, Thousand Oaks, CA USA; 14 Children’s Hospital Colorado, University of Colorado School of Medicine, Aurora, CO USA; 15 BDSRA Australia, Killarney Vale, NSW Australia; 16 BDSRA Foundation, Columbus, OH USA; 17 Beyond Batten Disease Foundation, Austin, TX USA; 18 NIHR Great Ormond Street Hospital Biomedical Research Centre, London, UK; 19 German Center for Child and Adolescent Health (DZKJ), Hamburg, Germany

**Keywords:** CLN3, Batten, NCL, Modified-Delphi, Vision loss, Neurodegeneration.

## Abstract

**Background:**

CLN3 disease, also called Juvenile Neuronal Ceroid Lipofuscinosis (JNCL), or Batten disease, is an ultra‑rare, neurodegenerative lysosomal storage disorder generally affecting individuals during the first decade of life. There can be a delay in diagnosis or misdiagnosis due to a lack of awareness, and when the most common presenting symptom of visual loss is attributed to more common conditions affecting vision.

**Methods:**

We used a previously published Expert Mapping Tool (EMT) to identify multidisciplinary professionals with diagnostic or clinical management expertise, as well as patient advocates with experience of CLN3 disease. A systematic literature review of published evidence using the Preferred Reporting Items for Systematic Reviews and Meta‑Analyses (PRISMA) guidance was conducted independently and simultaneously to develop key clinical care statements. Each statement was based on the strength of the evidence. The statements formed the basis of an international modified-Delphi consensus process using a virtual meeting platform (Within3). Experts were asked to agree or disagree with each statement and suggest any changes. Statements that reached a consensus of 75% or over are the guiding statements within this manuscript. The processes and manuscript have been independently assessed using the Appraisal of Guidelines for Research and Evaluation (AGREE II) criteria.

**Results:**

Thirty‑nine international experts from eight specialities were identified, including a patient advocate. Fifty‑three recommendation statements were developed covering eleven domains: General statements, Diagnostics, Clinical Recommendations and Management, Assessments, Social Considerations, Ocular Management, Epilepsy/Seizures, Nutrition, Respiratory Health, Sleep and Rest, and End-of-Life Care. Consensus was reached after one round of voting for all except three statements. The overall AGREE II score for developing these recommendations was 6.4, where 1 represents the lowest and 7 is the highest quality.

**Conclusion:**

Currently, there are no comprehensive clinical recommendations for CLN3 disease. These recommendations provide a comprehensive, evidence- and consensus‑based tool that can be used by all healthcare professionals involved in the management of CLN3 disease and other similar neurodegenerative conditions. The goal is to address the unmet clinical need for CLN3 disease management and complement other information available.

**Supplementary Information:**

The online version contains supplementary material available at 10.1186/s13023-026-04298-2.

## Background

The neuronal ceroid lipofuscinoses (NCLs), collectively termed Batten disease, are rare early‑onset progressive neurodegenerative disorders [1]. Historically, they were classified according to age at first symptom onset and histologically by the ultrastructure of auto-fluorescent storage material accumulating in lysosomes [2]. This heterogeneous group of disorders is now classified by the affected gene and clinical phenotype, incorporating age of onset and progression [2]. Up to thirteen different genetic forms and over 625 pathologic variants have been identified [3]. The focus of these recommendations is the most prevalent NCL form, CLN3 disease, previously called Juvenile Neuronal Ceroid Lipofuscinosis (JNCL, Batten disease, Spielmeyer-Vogt-Sjögren disease) [4, 5] (OMIM #204200), caused by biallelic pathogenic variants in the *CLN3* gene [2, 6]. CLN3 disease is an ultra-rare, autosomal recessive, neurodegenerative lysosomal storage disorder generally affecting individuals in the first decade of life, although first symptoms can appear at later ages [7, 8]. In around 70–85% of patients, the pathological variant is a homozygous approximately 1-kb deletion of two internal exons (exons 7 to 8) [9], which can cause more than 25 disease transcript variants with evidence suggesting that this results in protein isoforms that either lose function, retain partial function [10], or even gain a novel function [8, 11]. These patients are considered to have the ‘classic’ juvenile CLN3 disease phenotype. The classic phenotype is fairly homogeneous [114], but there is also phenotypic variability [12]. Around 20% of cases contain the 1-kb deletion and another variant on the other allele [3, 9, 13]; other cases do not carry the 1-kb deletion at all. Some of these patients have a later age of onset or more protracted phenotype, likely due to partially preserved function of the CLN3 protein.

**Fig. 1:**
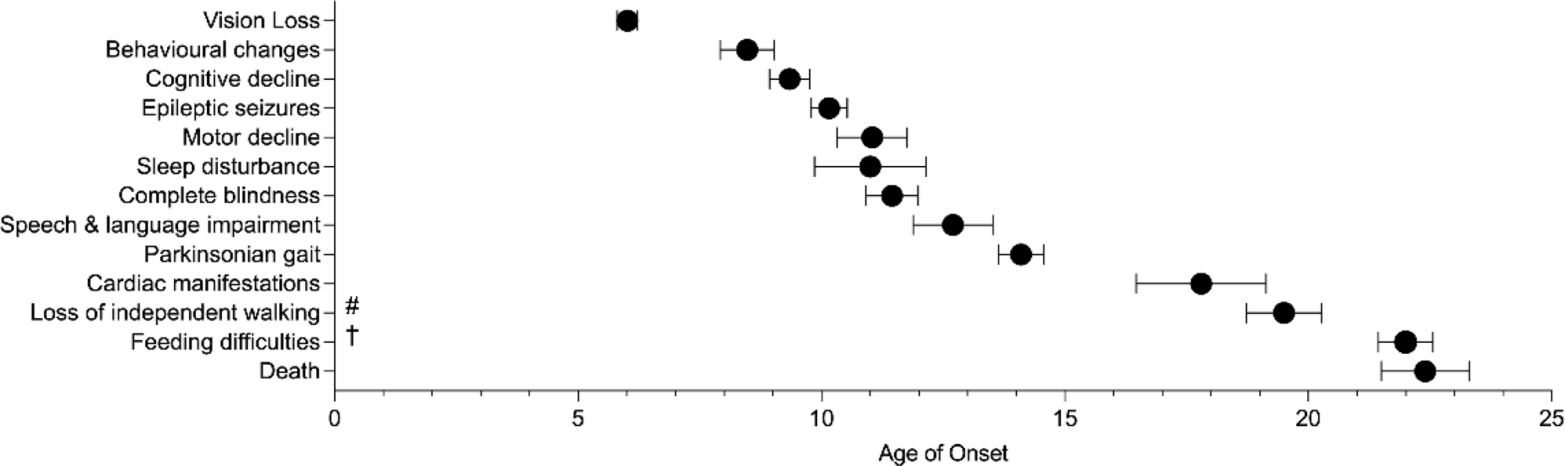
Timeline of disease progression in classic CLN3 disease. Timeline of weighted mean age and 95% confidence interval for the initial onset of the core clinical symptoms in CLN3 disease. # Unable to walk without assistance or daily use of wheelchair; † Requiring enteral feeding tube. Figure adapted and reproduced with permission from Ineka Whiteman et al. [22]

CLN3 is a 48kD lysosomal/endosomal transmembrane protein; its primary function remains unknown [14]. In the classic juvenile CLN3 disease [15], children present between four and eight years of age with vision impairment due to rapidly progressing retinal dystrophy with macular involvement [16–18], followed by behavioural changes, cognitive decline [19], epilepsy, and a movement disorder [17, 20] (Fig. 1). Motor dysfunction is most commonly progressive parkinsonism that leads to complete loss of ambulation in the later stages [21]. In addition to these neurologic symptoms, cardiac manifestations such as sinus bradycardia and left ventricular hypertrophy occur in later disease stages. Death usually occurs within the third decade of life [17].

There are individuals with CLN3 disease who are affected only by retinal dystrophy, which presents in childhood or as late as mid‑to‑late adulthood, or with a slower disease course than classic juvenile CLN3 disease [23].

Currently, there is no cure for CLN3 disease or any form of NCL disease, so treatment options are limited to symptom management and palliative care [24, 25].

These CLN3 disease recommendations were developed following the interest in the CLN2 disease guidelines published in 2021 [26]. Internationally agreed-upon recommendations, supported by an expert faculty, developed through a robust methodology and assessed by independent assessors, are essential. Clinical practice guidelines promote high‑quality and proactive care, reducing barriers to multidisciplinary management that arise due to lack of familiarity; this can mean the difference between ‘diagnostic odysseys’ or early diagnosis, no care or substandard care, and patients and their families across the world living a better quality of life (QoL) for longer, with fewer complications [27].

***Health Questions to Be Answered by These Recommendations***:

1. How can early identification and diagnostic pathways be improved for patients affected by CLN3 disease?
2. What care packages best help patients with CLN3 disease and their families?
3. What supportive therapeutic options are currently available, and what is the expert consensus on their appropriate use?
4. What are the key areas of unmet need and current knowledge gaps faced by clinicians and families affected by CLN3 disease?

### Objectives

Currently, there are no comprehensive clinical recommendations for the management of CLN3 disease. The rarity of this condition and extremely limited clinical experience in the broader medical community inhibits current consensus. Here, we aim to develop recommendations that provide guidance for the identification and clinical management of patients with CLN3 disease, independent of age and disease severity. These are expert-agreed practical recommendations. They can be used by healthcare professionals managing the holistic care of patients and serve as a point of reference for families and advocates, best placed to raise awareness of CLN3 disease among non-expert healthcare, social care and education providers, empowering all parties to support the management of individual patients. They can accelerate diagnosis and enable the timely provision of helpful therapeutic interventions worldwide [28].

### Methods and process

#### Expert Mapping Tool (EMT) development

A four‑stage process was used to identify global experts in CLN3 disease. While each stage of the process is time-consuming, the process removes selection bias. This EMT was previously used during the development of the CLN2 guidelines [26]. The first stage captures the highest input level in the current literature for CLN3 disease. PubMed was interrogated with predetermined search terms. The resulting publications were screened for relevance to CLN3 disease. All authors listed in each publication were sorted according to the number of appearances. Of these authors, those who appeared in two or more publications were selected and further ranked as follows: 2 publications (score 1), 3–5 publications (score 3), 6–14 publications (score 5), and more than 15 publications (score 6).

The second stage in the EMT determines the author’s Hirsch index (h-index) [29], which acts as a quantified guide to their total effectual output. Any search engine can be used (e.g. Scopus, Google Scholar Publish or Perish), although the same engine must be consistently used for all authors. Searches were conducted using the author’s last name and first name, and their profiles were found based on their occupation, middle initial, city, and country of residence. The h-index for each author was ranked as > 10 (score 1), 11–29 (score 3) or > 30 (score 5).

The third stage identifies individuals perceived as leading experts among families and advocates. Publicly available online information was scrutinised to identify experts who were, or had previously been, involved in patient organisation events or conference programmes during the previous five years. Search terms were CLN3, Batten disease, and Neuronal Ceroid Lipofuscinosis. Chairs and oral presenters were cross-referenced with the Batten Disease Family Association, UK (BDFA), Batten Disease Support and Research Association, USA (BDSRA), and other organisations focusing on CLN3 disease. Presenters, chairs and poster authors were tallied and ranked according to the number of appearances. Authors who appeared on less than three occasions scored 4; on three to four occasions scored 5; and on greater than five occasions scored 6.

The final stage identified those considered experts by their peers, whose expertise on CLN3 disease is called upon by the medical community. A search was conducted for all scientific meetings relevant to CLN3 disease during the previous five years (Appendix 1). The number of appearances was ranked as three to five (score 2) or six or more appearances (score 4).

The weighted scores of each stage were combined, creating a combined ranking of experts. Authors who did not feature in multiple rounds or scored less than ten were not considered. Animal experts were not considered for chair positions, and industry professionals were excluded. Currently practising and retired clinicians involved directly with patients affected by CLN3 disease were included to increase the diversity of opinion. The two chairs were proposed to be from different geographical regions to drive international collaboration, fill knowledge gaps, and further reduce bias. Complete data analysis for the EMT has been presented elsewhere [30].

#### Convening the steering committee

Following the implementation of the EMT, two chairpersons were identified. Together, they debated which specialities were needed to encompass all aspects of CLN3 disease management. The programme was led by an independent multidisciplinary Steering Committee (SC), recommended by the chairs, and was free from the influence of external stakeholders. The role of the SC was to validate the programme process, focus on project objectives, develop the clinical questions, and make recommendations that can benefit local clinical practice across a breadth of healthcare systems globally.

The SC were selected based on their involvement with patient organisations, which, combined with their academic output, covered the entire scope of the recommendations. A list of 22 SC experts from Europe (11), North America (9), and Asia (2) contributed to the development of these recommendations. These experts comprised patient advocates, a parent, paediatric neurologists, paediatricians, geneticists, neuropsychologists, a nurse, metabolic specialists, primary care specialists, ophthalmologists, ocular geneticists, palliative care physicians, pain specialists, and researchers. Further details, including competing interests, institutions, and contributions of each SC member, are listed within the declarations section of this manuscript.

#### Systematic literature review methodology

Parallel to the EMT and SC selection, independent systematic literature reviews were conducted by two medical writers, one internal and one externally contracted. Each literature search was first performed on the 16th of August 2021 through the Firefox or Google Chrome browsers, interrogating both PubMed and EMBASE databases, and updated on the 23rd of October 2023. Both searches used the same criteria (Appendix 2). All publications on humans and animals between 1997 and 2021 were included. Publications were excluded if the abstract did not directly relate to CLN3 disease, were narrative reviews, preclinical, or animal studies, or were conference proceedings, comments, errors, retracted or retired.

The rationale for the search criteria cut-off date was due to the spectrum of mutations being identified by Munroe et al. (1997) [9]. No filters were added for language or article type. Search strings incorporated Medical Subject Headings (MeSH)(PubMed), and Emtree (EMBASE): ceroid lipofuscinosis-neuronal, ceroid lipofuscinosis-neuronal juvenile type, and Batten disease. Free text keywords were defined based on Problem/Patient/Population, Intervention/Indicator, Comparison, and Outcome (P.I.C.O.) methodology to answer each clinical question [31, 32]. The results included systematic reviews, meta-analyses, descriptive observational studies, and interventional early‑phase non-randomised and open‑label clinical trials. The bibliographies from each publication were reviewed to ensure all additional relevant information was included. All SC members were invited to provide publications that the literature reviews may not have identified.

The abstracts of each publication were reviewed independently by each medical writer. Discrepancies in the decision to include or exclude a publication for data extraction were resolved during a virtual meeting between the medical writers. P.I.C.O. data was extracted by a full paper screening of each remaining publication by both medical writers to form the basis of the clinical statement development. Each publication selected for data extraction was assessed using the Oxford Centre for Evidence-Based Medicine (OCEBM) criteria [33]. General disease focus, genetics, diagnostics, clinical management and therapy information was collated. Study design, patient population (CLN3), interventions (all), comparisons (if appropriate), outcomes and the limitations of each study were also reported.

The chairs (JWM/AS) co-wrote the clinical care statement drafts based on these P.I.C.O. data. These statements were the basis of the consensus‑building process. An internal medical writer assessed each piece of literature linked to a particular clinical statement, using the Oxford Centre for Evidence-Based Medicine (OCEBM) criteria [33]. Independently, one chair and one SC member gave an OCEBM grade for each article and cross-referenced the grades made by the internal medical writer. The chair and SC member independently identified missing articles or evidence from each statement. Based on the three independent grades, an average grade was given to each article, followed by an overall evidence grade between all articles relating to each statement (see tables).

#### Consensus building: statement development meeting

SC members were invited to join a virtual meeting hosted by the Within3 online hosting platform [34], which was used to establish the SC’s communication preferences, biographies, conflict of interest statements, availability during the programme, and recommendations for further committee members. Within3 is a 24/7 virtual environment that allows stakeholders to interact according to their availability, while enabling the chairpersons to organise materials in one secure place, post feedback, and respond to questions.

In a second Within3 meeting, the drafted statements were uploaded for the consensus‑building phase of the recommendation development programme. The Within3 session has required resources, which must be opened and reviewed by each member to satisfy the requirements of the virtual meeting.

The main topics for developing these recommendations were drawn from management strategies for CLN2 disease published by Williams et al. (2017) [35] and also from the CLN2 guidelines published in 2021 [26]. These topics, our updated systematic literature reviews, and individual input from our SC ensured that no areas relevant to patient care were omitted.

Statement development was carried out over a 19‑day period. SC experts recorded their recommended changes to each statement, and the other SC members could respond to the feedback. Reminders were sent out over this review period to encourage participation and allow each member to contribute their expertise.

#### Modified-Delphi questionnaire: healthcare professionals

Once draft statements had been compiled using the Within3 platform, healthcare professionals (HCPs) from the wider community were recommended by SC members and invited to validate the statements. An anonymous voting process via live link was hosted by the collaborative research software Within3. The perspectives of all HCPs were collected. In the first round of voting, HCPs were asked whether they had ever managed a patient affected by CLN3 disease, if so, how many; whether their understanding of the English language was sufficient to complete the survey, their primary role; their main area of expertise; length of time in practice; type of primary practice and in which country; whether they had previously been involved in guideline development. The goal was to collect over 60 responses from at least six specialities responsible for caring for patients with CLN3 to ensure adequate independent input to each statement.

Each statement was graded via a Likert‑type scale of 1–10, where 1 was a total disagreement, and 10 was a strong agreement. Consensus was defined as ≥ 75% agreement or more on each statement, as per a systematic review, that found 75% to be the median threshold to define consensus for Delphi studies (range 50%-97%) [36]. Where consensus was not reached, the chairs reviewed the statement. Where simple semantics were required, the statement was changed. A subject matter expert was invited to present the topic if the statement presented polarising views. Statements reaching consensus have been included in this manuscript unchanged. The survey was left open for eight weeks to collect as many responses as possible.

#### Quality assessment

The quality of the development and reporting strategy for the recommendations was assessed by two independent reviewers using the AGREE II instrument [37]. This validated tool evaluates the methodological quality of clinical practice guidelines. The tool is intended to improve the comprehensiveness, completeness and transparency of reporting. The AGREE II checklist comprises 23 items divided into six domains: Scope and Purpose, Stakeholder Involvement, Rigour of Development, Clarity of Presentation, Applicability, and Editorial Independence. Each item was rated on a scale from “not met” [1] to “fully met” [7]. Suggested amendments were made where possible, and a second round of assessment was carried out. Combined scores for each domain were calculated using the following equation: (obtained score-minimum possible score) / (maximum score possible) x 100. An overall average score was calculated from a maximum value of 7.

## Results

### Expert Mapping Tool (EMT)

The EMT identified 834 professionals who were sequentially approached after ranking them according to their weighted scores until two could commit to participation in this programme as chairs. We secured one chair from the USA and another from Europe (Germany) to provide a degree of global relevance. The highest‑scored expert was 18, and the lowest was 10. The two selected co-chairs had individual scores of 18 and 16.

#### Systematic literature review

Results were reported in accordance with the Preferred Reporting Items for Systematic Reviews and Meta-Analysis (PRISMA) statement [38]. The systematic review conducted by the contracted medical writer identified 5972 publications. After removing duplicates, 1344 were screened, and a further 1099 met the exclusion criteria. Qualitative analysis and Population, Intervention, Comparison and Outcomes (P.I.C.O.) summaries were completed for 245 publications (Appendix 3). The systematic literature review conducted by the internal medical writer identified 6006 publications. After removing duplicates and those that met the exclusion criteria, 1470 were screened, and 243 were included in the qualitative analysis (Appendix 4). The qualitative analyses from both literature reviews were pooled into one document. P.I.C.O. summaries were possible for 240 publications (Appendix 5); the remaining five were not in English.

Due to the global COVID-19 pandemic, the SC members’ availability disrupted this programme’s progress. As such, a further analysis of the literature was conducted on the 26th of October 2023, to capture any updates in CLN3 that had been published. The same previous search strategy was used, using PubMed, which identified 275 publications, 182 of which were duplicates. Of the remaining 93 publications, 15 were included in the context for discussion in the appropriate sections (Appendix 6).

#### Consensus building: statement development meeting

During the Within3 stage of the consensus process, 2078 comments were posted by the SC. The comments were distributed through the twelve topics as follows: Background (87), General Statements (173), Diagnostics (314), Clinical Recommendations and Management (322), Assessments (350), Social considerations (281), Ocular Management (113), Epilepsy / Seizures (79), Nutrition (111), Respiratory Health (71), Sleep and Rest (68), End-of-Life Care (109).

#### Consensus building: modified-Delphi questionnaire

All 39 experts who responded to the questionnaire considered their English language proficiency adequate to complete the survey. The lowest number of respondents to any question was 33, and consensus ranged from 63% to 99%. Consensus was reached after one round of voting for all except three statements. A second round of Delphi was attempted but failed to reach the participation criteria of at least 60 responses from six specialities.

#### Appraisal of guidelines for research and evaluation (AGREE II assessment)

##### Recommendation statements

Recommendation statements were developed based on the results of the systematic literature review, which revealed twelve different topics of clinical focus. The topics were General Statements, Diagnostics, Clinical Recommendations and Management, Assessments, Social Considerations, Ocular Management, Epilepsy/Seizures, Nutrition, Respiratory Health, Sleep and Rest, and End-of-Life Care.

### General description of CLN3 disease and statements (Table 1)

In over 80% of cases, symptoms caused by CLN3 disease usually arise between four and eight years of age, initially presenting as visual impairment due to rapidly progressive and severe retinal dystrophy with early macular involvement, with degeneration of the retinal rods and cones [5, 20]. The period between the onset of visual loss and neurological symptoms ranged from 1 to 9 years [39]. A slowing of walking speed manifests between 7 and 8 years, progressing to a parkinsonian gait [40]. Cognitive impairments typically begin early, characterised by an initial slowing of cognitive growth, followed by a plateau and then a loss of previously attained abilities; however, this pattern is not uniform across all areas of skill. Most remain aware of their surroundings into the second decade of life [17, 41]. Generalised, complex partial, or occasionally myoclonic seizures develop between 10 and 11 years of age [42]. In the middle teenage years, a significant proportion of patients experience progressive cardiac involvement. The pathology is associated with repolarisation disturbances, ventricular hypertrophy, and sinus node dysfunction [20]. Anxiety and fearful behaviours are common as the disease progresses [43]. In later stages of the disease, fearful episodes may be triggered by unexpected sounds, changes in position and manual handling, or separation from a parent or caregiver [44]. Death usually occurs in the second or third decade of life [45]. Females tend to have an earlier onset of vision loss but may experience a later onset of behavioural problems [45]. Death is usually earlier for females than for males [45, 46].

Most individuals with CLN3 disease have a characteristic phenotype, although variations exist and have been described as “classic”, “vision‑loss only”, “attenuated‑course”, and “delayed‑onset” in the literature [47, 48]. Intrafamilial variability in clinical progression exists [19, 49], especially in compound heterozygotes, who show greater phenotypic variation than common deletion homozygotes [12, 50]. Compound heterozygotes may have atypical phenotypes, including a milder course of disease [51], but most have a classic phenotype [52]. Common deletion homozygous phenotypes may vary in the rate of progression of psychomotor regression; however, age at onset and progression of visual loss are comparable in the majority of cases [53]. Different transcript variants and epigenetic processes may play a role in the differential expression of the mutated gene, post‑translational modifications [54], or certain modifier genes that affect the clinical course of CLN3 [12]. The relationship between pathogenic variants and phenotypes is not entirely understood [55]. These recommendations aim to encompass the spectrum of CLN3 disease without distinction among phenotypes. The lack of understanding of aetiology and pathogenesis means that disease-modifying treatment options for CLN3 disease remain limited [56].

Four statements were developed to support the general description of CLN3, with all statements reaching a consensus.

**Table 1:** General statements regarding CLN3 disease, statements and consensus data

Statement	Responders	Evidence Level	Consensus %
Juvenile CLN3 disease is one of the most prevalent forms of Batten disease at the time of guideline development. Symptoms of visual loss begin between the ages of 4 and 8 years in > 80% of cases, and symptoms evolve to include dementia (including cognitive regression and mood and behavioural disturbances), seizures, motor deficits and, in a significant number of patients, cardiac conduction problems.	38	5	87
CLN3 disease is also commonly known as Juvenile Neuronal Ceroid Lipofuscinosis (JNCL) since initial symptoms often appear during this stage of life. The classical term Batten-Spielmeyer-Vogt disease has a historical value. Batten disease refers to the family of disorders arising from defects in the Ceroid Lipofuscinosis Neuronal (CLN) genes, with subtypes classified according to the NCL gene affected (e.g. mutations in CLN1, CLN2, or CLN3 gene gives rise to CLN1 disease, CLN2 disease and CLN3 disease, respectively).	35	4	91
Approximately 75% of CLN3 disease alleles have the same “common” 1 kb intragenic deletion.	34	4	85
Phenotypically, CLN3 disease is often heterogeneous in symptom profile, age of onset and pattern of progression. These guidelines aim to cover the whole spectrum of the disorder, with no distinction between varying phenotypes.	33	4	78

### Diagnostics (Table 2)

The diagnosis of CLN3 disease is often delayed, sometimes up to several years [57–59]. Families frequently describe their experience as a ‘diagnostic odyssey’; a common burden across rare diseases. Lengthy, uncertain and stressful diagnostic journeys significantly impact the emotional, psychological and physical health of caregivers [60]. With the development of a unified diagnostic algorithm and commonality of diagnostic steps, misdiagnoses and underdiagnoses can be minimised or overcome [61, 62].

Historically, an important diagnostic work‑up involved structural analysis of lymphocytes and ultrastructural analysis of skin biopsies via electron microscopy (EM) to test for the presence of lysosomal storage material [63] alongside clinical features [61]. EM shows characteristic membrane-bound lipopigments in the cytosomes, appearing as tightly bound, parallel, paired dense lines resembling fingerprint profiles [64] or vacuolated lymphocytes [65–67]. Peripheral blood smears show that between 5% and 10% of lymphocytes from patients with CLN3 disease have vacuolisation. Lysosomal‑associated membrane protein‑1 (LAMP-1) expression is observed in all lymphocytes (CD4 and CD8) of CLN3 patients, even in the absence of vacuolisation. LAMP-1 expression is more pronounced in T-cells of patients with classic and attenuated CLN3 disease, as well as in B-cells of patients with retina-only CLN3 disease [68]. Vacuolisation and LAMP-1 expression increase with phenotypic severity.

Microscopic techniques remain important as supportive diagnostic tools in cases when the pathogenicity of novel sequence variants in the *CLN3* gene is unclear [69]. Next‑generation sequencing (NGS), *with* micro-array deletion duplication testing, is now considered the “gold standard” for diagnosing CLN3 disease.

A CLN3 disease diagnosis should be considered in all children under the age of 10 years presenting with rapid best‑corrected bilateral visual loss [57], with or without photophobia and night blindness [70]. The early ocular symptoms can be non‑specific [71], leading to misdiagnoses by ophthalmologists as Stargardt disease, retinitis pigmentosa, or rod‑cone dystrophy [39]. The electroretinogram (ERG) in symptomatic patients may be typically electronegative, with a diminished b-wave amplitude [72] or no recordable rod‑mediated activity [73]. Around 20% of patients also present with clinically evident maculopathies on fundoscopy and optical coherence tomography (OCT) around the time of diagnosis [71, 74].

Attenuated and atypical CLN3 disease cases may also first present to adult ophthalmology clinics, where affected individuals present in their 20s [73] or older [75, 76]. Neurological symptoms may be absent, presenting only with bilateral retinal degeneration, with severely reduced or undetectable rod and cone ERG responses. In these adults, initial misdiagnoses include retinitis pigmentosa [75], autoimmune or paraneoplastic retinopathy [76], or fundus albipunctatus [77]. Characteristic vacuolated lymphocytes and ‘fingerprint’ or ‘curvilinear’ profiles may be seen, albeit at lower concentrations than classic juvenile CLN3 disease [73, 75]. Ophthalmologists play a vital role in the early diagnosis and monitoring the progression of CLN3 disease [78]. As such, targeted education initiatives should be a priority for patient advocacy groups and relevant healthcare networks to improve knowledge and awareness in the ophthalmology community, particularly the paediatric subspecialty [39]. Therefore, CLN3 should also be considered an aetiology in adults with progressive neurological decline and a history of bilateral vision loss [79–82].

Electroencephalograms (EEGs) are often normal before 9 years of age, with progressive background abnormality and an increase in paroxysmal activity [83]. The neuroradiologic picture is normal in 25% of cases. With more advanced disease, MRI imaging shows evolving cerebral and cerebellar cortical grey matter atrophy, and often non‑specific periventricular white matter changes in the temporo‑occipital regions [84]. Calcification in the transitional area between grey and white matter in the temporal lobes has also been reported [67]. Cerebral leukoencephalopathy is present in around 45% of patients, and 36% show thalamic T2W-hypointensity [85].

Significant differences in quantitative T2 values within the white or grey matter in the thalamus have not been reported in CLN3 disease, in contrast to CLN2 disease. T1‑weighted, 3-dimensional magnetic resonance (3D-MR) images using voxel‑based morphometry show a significant reduction in grey matter volume in the dorsomedial part of the thalami.

Additionally, the volume of white matter was significantly decreased in the corona radiata, suggesting that these two areas may have a role in the pathogenesis of CLN3 disease [86]. In adolescent CLN3 disease patients, the annual rate of grey matter loss is as high as 2.4% [87]. Positron emission tomography (PET) scans reveal a mild reduction in striatal dopamine D1 receptors, but not in D2 receptors; however, the contribution of these changes to extrapyramidal symptoms remains unknown and warrants further investigation [88].

Autophagic vacuolar myopathies have been described in association with mutations in *CLN3* [65, 89], in attenuated, classic, and non‑attenuated CLN3 disease. Muscle biopsies reveal features of lysosomal pathology, with autophagic vacuoles and sarcolemmal features. Hence, genetic testing for all known pathogenic variants in *CLN3* should be considered in cases of autophagic vacuolar myopathy [90, 91].

Cardiac MRI findings show abnormal storage material within the myocardium in CLN3 disease [92].

Advances in translational research would increase disease awareness, reduce diagnostic delays and facilitate treatment developments. However, epidemiological data in the rare disease domain are lacking. Early or even prenatal diagnosis allows families to receive appropriate family planning [64], counselling and provision of educational and social support [16]. In future implementation of CLN3-specific disease-modifying treatments, early diagnosis will be crucial for optimal care.

Seven statements were developed to support the diagnostics of CLN3, with six reaching consensus. One statement fell slightly below the agreed 75% threshold for consensus (73%), which was due to differences in local second-line screening protocols.

**Table 2:** Diagnostic statements and consensus data

Statement	Responders	Evidence Level	Consensus %
Timely diagnosis is desirable and crucial to avoiding lengthy ‘diagnostic odysseys’ and allow genetic counselling.	33	4	99
With the approval of CLN3-specific treatments in future, early diagnosis, including presymptomatic diagnosis, will be paramount.	33	4	98
When a child presents with unexplained rapidly progressive bilateral vision loss, NCL should be considered in the differential diagnosis. The ophthalmologist should be aware of this rare entity, and targeted clinical education is desirable in this field.	33	4	95
Identification of two disease-causing alleles is the ‘gold standard’ for NCL diagnosis. Light microscopy analysis of lymphocytes and ultrastructural analysis of tissue containing neurons can also be helpful tools for identifying new variants and where rapid genetic testing is unavailable.	33	4	85
While genetic testing presently appears to be the most common first-line diagnostic approach, in the circumstances where CLN3 gene mutations of unknown significance are detected, skin biopsy can also serve as an important second-line tool for confirming the pathological presentation of CLN3 Disease. i.e. skin biopsy and lymphocyte vacuolisation as second-line confirmation.	32	4	73
CLN3 disease should be considered in adults presenting with visual impairment when the history, ocular and systemic examination are supportive.	33	5	76
The diagnosis of CLN3 disease can be confirmed genetically through next-generation analysis, guiding future treatment and prognostic evaluation.	33	3	86

### Clinical recommendations and management (Table 3)

All patients with a suspected diagnosis of CLN3 disease should be promptly referred to the nearest healthcare facility with genetic testing capabilities and resources to conduct the recommended battery of diagnostic assessments and investigations. Ideally, referrals should be made to a centre specialising in NCL care. NCL centres are available in several regions. Where access to these resources is unavailable, local providers should seek advice from specialist NCL centres.

It is crucial for parents or caregivers, as well as the affected individual themselves, to have trust in their healthcare team from the outset and throughout the Batten disease journey. When delivering the diagnosis, clinicians should provide relevant, accurate and up‑to‑date information at a level and format appropriate for the parent or caregiver [93]. Ideally, a genetic counsellor should be present when the result of genetic testing is delivered. Psychological support should be offered to all family members at diagnosis, during the course of the disease, and at times of significant changes, including the death of a child [94]. Diagnosis of a progressive illness and the lived experience of each step of the illness journey have a significant emotional impact on the individual and the whole family. Having access to a peer support group can be a source of comfort and empowerment [95].

Families should also be given the contact information of the relevant NCL patient support organisations. Patient support or patient advocacy organisations are non-profit groups, often founded and run by families affected by one of the NCLs. Patient organisations are equipped with vast experience in the lived experience of NCL. They can offer practical support, education and resources, and vital peer support to assist families at all stages of the Batten disease journey, from diagnosis to bereavement [93]. Some patient groups also fund and actively drive research toward discovering treatments and cures, and help advance care and management strategies toward improved QoL. Their interactions with the scientific community, clinicians, biotechnical industry partners and policymakers put patient groups well‑positioned to raise awareness, disseminate information, influence policy, and be at the forefront of therapeutic research and development [94].

Ongoing communication and care coordination by a multidisciplinary team (MDT) of paediatricians, neurologists, ophthalmologists, geneticists, neuropsychologists and psychologists, nurses, physiotherapists, palliative care specialists, and other stakeholders, such as social and educational services, is essential for optimal holistic care [26]. A responsive team that adapts to the changing needs of the patient and their family has a significant positive impact on the QoL for families [95].

The clinician responsible for coordinating care should have knowledge of CLN3 disease pathology and the disease progression. Skilled clinical evaluation of adaptive function is required throughout the course of disease progression in order to assess and respond to changing needs for support in accomplishing activities of daily living and to maintain community engagement. Clinical rating scales may be useful to evaluate disease burden and progression [96]. Established disease‑specific rating scales include the Unified Batten Disease Rating Scale (UBDRS) [97] and the Hamburg JNCL scale [98], which are widely used to evaluate and monitor disease progression in research settings [99]. They may also be useful for assessing progression in routine clinical care.

Depending on their ability to tolerate clinical procedures, some individuals with CLN3 disease may require general anaesthesia (GA) for diagnostic procedures, gastrostomies, and ophthalmic diagnostic testing. The majority of GAs are managed without complications. However, postoperative bradycardia, hypotension and hypothermia have been reported [100]. Thus, careful neurological and cardiac function assessment should be carried out before GA. Short‑acting anaesthesia is recommended, and in patients with severe epilepsy, anti-seizure medications (ASMs) should be continued with EEG monitoring if warranted [101].

Gender differences have been described in the rate of progression of motor symptoms in CLN3 disease [102]. The impact of disease on physical aspects of health‑related quality of life (HRQoL) scores is worse for females than males, with an average later onset of symptoms [46] but earlier loss of independent ambulation, earlier placement of a gastric tube and death at a younger age, suggesting that the disease course for females unfolds more rapidly [102]. Behavioural and psychiatric symptoms do not differ systematically between genders [103]. Hyperandrogenism, characterised by acne, early menarche, signs of anovulation, and hirsutism, has been reported in females with CLN3 disease [104].

The mainstay of current therapy for CLN3 disease is symptomatic, but symptomatic care does not stop the progressive course of the disease. Clinical management includes epilepsy medication to control seizures, supportive therapies such as speech therapy, physiotherapy, feeding tubes or gastrostomies, non-restrictive use of common painkillers when needed, and management of other symptoms as they arise. Some evidence exists for the use of dopaminergic drugs to manage parkinsonian symptoms, but more study is needed [105].

Given the heterogeneity of CLN3 disease, gathering robust and systematic longitudinal natural history data of the disease, including cognitive function and vision loss, can help inform management and educational decisions in future need planning. Should future interventional therapies become available, longitudinal natural history data may serve as an effective comparator measurement of efficacy [106].

Eight statements were developed to support the clinical recommendations and management statements of CLN3 disease, with seven achieving consensus. One statement in this group regarding gender differences was under the agreed 75% threshold for consensus (63%). This is likely due to different clinical experiences among the experts but no definitive data to drive concurrence.

**Table 3:** Clinical recommendations and management, statements and consensus data

Statement	Responders	Evidence Level	Consensus %
All patients with suspected CLN3 disease should be provided information about the centres with expertise in diagnosing and managing patients with NCL disorders and referred to an NCL expertise centre if at all possible. If access is challenging due to geographic or other barriers, local providers should seek input from an NCL centre.	33	4	86
Parents should be directed to a medical provider who has knowledge of disease pathology, progression and experience in treating children with CLN3 disease for the first consultation. Ongoing communication and education should be provided to optimise patient outcomes and parental support.	33	4	92
Following diagnosis, patients should be referred to an NCL patient support organisation in their country/region and, through these, encouraged to connect with other NCL families and communities. A number of Batten disease patient support groups are active worldwide and can offer valuable peer support and opportunities for information and resource sharing	33	5	90
Gender differences may be seen in the pathophysiology of CLN3 disease patients. Hyperandrogenism is common, with early menarche and signs of anovulation.	33	3	63
Patient care should be provided by a multidisciplinary team of local care, paediatricians, neurologists, ophthalmologists, or other specialists with clinical experience in CLN3 disease. Ongoing communication and coordination of care among providers is paramount.	33	4	95
Patient-centred care is critical for CLN3 disease management, and a multidisciplinary team (MDT) is advised where possible to manage the diverse range of disease manifestations.	33	4	96
Families impacted by CLN3 disease should have access to a MDT to provide emotional and psychological support around caregiver burden, coping, and anticipatory grief. Paediatric and adult palliative medicine teams may be available to help support parental decision-making, especially in emotionally complex circumstances. Also, emotional support and social services/community support must be offered to families by centralised services, if possible.	33	3	93
Families should be offered psychological support/counselling and information about relevant patient organisation contacts following a diagnosis and over the course of the disease process as appropriate, as their support needs may change over time.	33	5	98

### Assessments (Table 4)

After obtaining verbal consent from the parent or caregiver, a comprehensive medical history and multisystem assessments should be conducted at the first clinic visit. Baseline physical, cognitive, behavioural, and neurological manifestations, functional ability, and disease burden should be documented to establish a baseline from which to assess the natural historical progression of the disease. A basic staging system can be used to categorise the progression of CLN3 disease [107]. Stage 0 – pre-symptomatic individuals with genetically confirmed CLN3 disease; Stage 1 – vision loss; Stage 2 – onset of seizures; and Stage 3 – loss of independent walking [108].

The well-established Hamburg JNCL scale is used to quantitatively assess motor and language function, as well as epilepsy and vision, to evaluate disease progression [98]. Although this scoring system only contains four items, with four scores, it demonstrates good statistical reliability [109]. Moreover, it can be used both retrospectively and prospectively and does not require specific training of the rater.

The validated Unified Batten Disease Rating Scale (UBDRS) is a disease-specific rating scale that assesses disease severity in four domains: physical impairment, behaviour, seizures, and functional capacity [110, 111]. The UBDRS helps evaluate the severity and rate of progression and to guide clinical interventions. In contrast to the Hamburg JNCL scale, the UBDRS is to be used prospectively, and raters should undergo specific training prior to its use. The physical subscale follows a quantifiable linear decline following symptom onset, which correlates with functional capacity and age but not with gender or genotype [52, 112]. Similarly, the seizure and functional capability scores correlate with age, whereas the behaviour score does not [113]. Prospective longitudinal examinations can be challenging; however, the UBDRS is designed for the visually impaired and those with speech decline and progressive dementia, where verbal responses are not always possible [114]. The UBDRS has excellent inter-rater and statistical reliability when used by trained raters [109–111].

Other assessments can take place in conjunction with the UBDRS. As noted above, adaptive function should be evaluated periodically to best quantify and respond to affected individuals’ needs for support in completing activities of daily living; this should entail the use of a psychometrically validated measure. As one example (though other standardised measures of adaptive function exist), the Vineland Adaptive Behaviour Scales, Third Edition (Vineland-3) can be completed via interview or questionnaire by an informant (e.g., parent) who knows the person well. The Vineland-3 Adaptive Behaviour Composite (ABC) Standard Score describes performance in relation to same-age peers across broad domains of communication, socialisation, and daily living skills. Separately, gross and fine motor skills comprise a Motor domain. The Vineland-3 ABC score is associated with the UBDRS Capability score and a composite score derived from performance on neuropsychological tests of vocabulary, verbal reasoning, verbal fluency, and sentence repetition [115].

Cognitive ability should also be evaluated at baseline and on a routine basis (at least annually, or more frequently as clinically needed). This information can guide school services and help establish realistic expectations for a child’s learning capabilities. Due to the rapid and progressive vision loss in CLN3 disease, it is necessary to use tasks that can be administered verbally and require only an oral response from the affected individual. In a cross-sectional analysis, the verbal IQ (VIQ) score from the Wechsler Intelligence Scales demonstrated a strong correlation with both the Vineland-3 ABC-SS and the UBDRS [116], i.e., verbal IQ decreased in association with lower adaptive function skills and worse physical function. Other psychometrically validated measures of cognitive ability can also be used to assess individuals with CLN3 Batten disease. Beyond the broad domain of IQ, cognitive assessments should include measures of attention, memory, and language [106, 116–118]. For example, working memory is impaired at an early stage of the disease [118]. Of note, standardised modifications in the administration of cognitive assessments may be required for children with visual impairment [117].

Because of the preponderance of psychiatric symptoms experienced by many individuals with CLN3 disease [119], routine evaluation of mood and behaviour is also indicated. Most research in CLN3 has focused on the Child Behaviour Checklist (CBCL), a standardised parent-proxy questionnaire to assess mood, behaviour, and psychiatric functioning in relation to same-age peers. If present, hallucinations, depression, or repetitive behaviours may not always correlate with disease progression, but nonetheless likely have a negative impact on patient and family QoL.

One study, using the 6-minute walk test (6MWT), showed that motor function impairment begins early after the diagnosis of CLN3 disease [120], but may be influenced by visual impairment. Quantitative spatiotemporal gait analysis showed that gait speed slows, the base of gait widens, and the time in the stance position increases [21]. 6MWT continuously declines with age and increasing UBDRS scores, correlating with disease progression [121].

Preliminary data from a longitudinal study have shown that 60% of patients develop cardiac pathology in the second decade [92]. Storage material accumulates in the valves, the conduction system, and the myocardium, causing myocardial hypertrophy and dilation of the ventricles [122]. While clinical symptoms are not always apparent, pathology shows repolarisation disturbances showing deeply inverted T waves and sinus node dysfunction that leads to bradycardia [123]. With increasing age, the heart rate is significantly reduced due to a decrease in sinus node automaticity related to a progressive accumulation of storage material in the sinus node [124]. Sinus node dysfunction can be successfully treated with a permanent dual‑chamber pacemaker; however, the decision for this intervention should take into consideration the patient’s prognosis and family priorities and consent [125–127]. There is a clinical need for regular cardiac evaluation.

Although the first symptoms of CLN3 disease may begin around four to eight years of age, conventional MRI imaging [84] and electroencephalograms are often normal in children under ten [83]. With increasing age, quantitative and visual analyses of electroencephalograms (EEGs) reveal background abnormalities and increases in paroxysmal activity.

High-density electrophysiological recordings can objectively measure sensory‑perceptual and cognitive processing. Brima et al. used an auditory evoked potential research paradigm to explore possible sensory processing biomarkers for CLN3 disease. The authors reported subtle findings for atypical auditory sensory memory in individuals with CLN3 disease that were correlated with age (i.e., more pronounced atypical processing with increasing age), a proxy for disease severity [128].

Grey matter atrophy due to neuronal loss is a feature of CLN3 disease progression [84]. Longitudinal analysis of the supratentorial cortical grey matter volume is a sensitive, quantitative parameter for assessing disease progression in all stages of CLN3 disease [84]. The pattern of storage deposition is most pronounced in neurons and macrophages within hippocampal regions CA2, CA3, and CA4 of the hippocampus [129]. Hippocampal volume decreases by around 3.3% annually, and the whole brain by 2.4% [130]. N-acetyl aspartate and glutamine/glutamate/gamma‑aminobutyric acid levels in the parietal grey matter decrease with age and, therefore, may be valuable indicators of CLN3 disease progression or a measure of treatment response [131]. Cortical grey matter volume exhibits a uniform decrease (4.6% annually) and strongly correlates with age [84]. It may be the most sensitive MRI parameter as an outcome measure in CLN3 disease in response to future therapies. Diffusion tensor imaging (DTI-MRI) reveals globally decreased anisotropy and increased diffusivity, most pronounced in the corona radiata and posterior thalamic radiation [132]. A decrease in structural brain connectivity networks, as measured by DTI-MRI, correlates with disease severity scores and symptomatology [133]. PET shows a reduced uptake of [^18^F] Fluorodopa in the putamen, which corresponds to disease severity, indicating that dysfunction of dopaminergic nerve endings contributes to extrapyramidal symptoms [134].

Longitudinal MRI analysis shows distinct pyramidal and interneuron degenerative patterns as well as microglial and astrocyte activation in the NCLs [129].

The development of quantitative markers of cortical volume, in combination with clinical markers, is needed to measure disease progression using cross‑sectional and longitudinal data for research [135].

Patients with CLN3 disease manifest many psychiatric symptoms with disease progression. Some psychiatric symptoms may improve with medications, especially anxiety, but caution should be used to avoid worsening the parkinsonism that is characteristic of CLN3 disease [136]. Careful evaluation of these symptoms by standardised methods should be undertaken before initiating treatment to optimise drug choice and dose [119]. There is an observed decrease in the density of dopamine transporters in the putamen [137], suggesting dopaminergic drugs such as levodopa offer a potential management strategy for Parkinsonian symptoms [105].

Ophthalmology follow‑up is crucial in the early diagnosis and follow‑up of CLN3 disease progression [78]. Supporting the child in learning visual aid techniques, including the use of a white cane, a sighted guide, zoom text, magnification and Braille, in the early stages may be beneficial. Still, there will be variation in the acquisition of skills and their retention, depending on the timing and rate of cognitive decline [114]. Differentiating features of CLN3 disease compared to the more commonly diagnosed Stargardt disease include a more extensively affected retina, the absence of subretinal flecks, severe colour vision abnormalities, and abnormal electroretinogram (ERG) responses [7]. Other ophthalmic diagnostics, such as OCT, fundus autofluorescence, and visual field testing, are also helpful if the child can participate in or tolerate them. Ophthalmic manifestations increase in severity with age and correlate with neurological symptoms. The Hamburg CLN3 disease ophthalmic rating scale serves as an objective marker for monitoring the degree of ocular severity [138].

In conjunction with the previously mentioned severity scales, observer‑reported outcome measures (obsRO), completed by caregivers of affected individuals, evaluate symptoms, function, and well-being, which can serve as powerful indicators of disease progression. ObsROs collect longitudinal data on elements meaningful to families (vision, seizures, language, dementia, mobility, and behaviour) [139, 140]. These outcomes monitor the degree to which changes in individual symptoms contribute to overall disability [141] and the burden on families concerning caregiving, education and healthcare [142].

Individualised educational plans should be implemented using supports, services, and accommodations to maintain engagement in academic activities and participation in real‑life activities [143]. These strategies aim to maintain acquired skills, reduce frustration and enhance QoL [5, 41]. Annual re-evaluation is recommended to monitor for changes in cognition and academic function; more frequent evaluation may be considered on an ad hoc basis to respond to emerging or worsening symptoms.

Nine statements were developed to support the recommendations for clinical assessments, with eight reaching consensus. One statement, on the use of the UBDRS in clinics, was just under the agreed 75% threshold for consensus (74%). The UBDRS is a research tool that has been applied in some, but not all, clinics. We think this did not reach consensus because of the different experiences of users.

**Table 4:** Assessments, statements and consensus data

Statement	Responders	Evidence Level	Consensus %
CLN3 disease patients may have cardiac conduction abnormalities in the second decade of life; therefore, regular cardiac evaluation should be considered from 15y onwards (echocardiography, ECG) or earlier if symptomatic.	33	4	86
The elements included in the UBDRS can be used to guide clinical assessments, but the performance of the full UBDRS is not needed in routine clinical assessments.	33	3	74
Following a diagnosis, with the consent of the parent/caregiver, a comprehensive medical history and multi-system evaluation should be conducted in order to set a baseline for assessments, not limited to physical and neurological manifestations of disease, functional ability and disease burden.	33	3	89
General well-being and growth are monitored in line with good clinical practice locally (e.g. growth might be assessed during the school’s annual medical exam), changes in vision are detected (e.g. by a specialist teacher) so that VI needs can be met, educational goals and interventions are reviewed at least every six months by specialist teaching staff, and symptoms and medication are kept under regular clinical review by an appropriate clinical expert 6–12 monthly as necessary.	32	3	87
MRI of the brain may be useful in the diagnosis and management of patients with CLN3 disease, although if the genetic diagnosis has already been confirmed, neuroimaging may not be required routinely.	32	3	79
Evaluation by an ophthalmologist is recommended, with appropriate follow-up if indicated. Ongoing functional vision assessment by an ophthalmologist, optometrist or specialist VI teacher may help in the early stages of disease in order to support the child with appropriate visual aids, mobility and Braille training as vision deteriorates further.	32	4	96
Annual or more frequently if needed, caregivers-reported outcomes are recommended to capture disease impact on patients and their families.	31	4	84
A similar statement but with a clarification and specification: specific therapies should be based on the needs of the individual.	32	5	88
There is some evidence for the use of dopaminergic drugs in the management of parkinsonian symptoms. Antidepressive and antipsychotic drugs can be helpful for behavioural symptoms in low or moderate doses but are often associated with exacerbation of parkinsonism and a higher risk for extrapyramidal side effects.	32	4	76

### Social considerations (Table 5)

Clinicians should be mindful that at diagnosis, the family have likely experienced a considerable period of ‘diagnostic odyssey’ and lengthy periods of uncertainty, with no definitive answer, diagnosis, or misdiagnosis, while watching their child deteriorate. This process causes extreme distress, with varying reactions to this devastating diagnosis. Families should be provided with the most up‑to‑date, accurate information and be encouraged to ask questions. Signposting to relevant patient advocacy associations is recommended for all family members [94]. As the disease progresses and complex care needs increase, there are significant emotional and psychological consequences for the affected individual and the family. Behavioural and emotional changes in CLN3 disease include verbal and physical aggression, self‑injury, anxiety and depression, and hallucinations [144]. Although these manifestations do not always correlate with disease severity, they can be a significant burden in daily life and negatively impact the QoL for the individual and family [145]. The mechanism by which the disease leads to specific behaviours and emotions is poorly understood and varies from patient to patient. Some data suggest that anxiety may be linked to deterioration in vision and mobility. Decline in cognitive and language skills may lead to frustration and anger. Additionally, changes in mood and behaviour are an inherent component of the paediatric dementia experienced in CLN3 disease. Physical and cognitive decline both lead to sadness in the affected individual [144]. There is commonly considerable disruption to family life, with siblings often unintentionally sidelined and romantic relationships challenged [146]. The negative impacts of these adversities can be mitigated by joining a parent advocacy community and receiving responsive, proactive social and healthcare services, including supportive counselling for parents and siblings [95, 144].

Individuals with CLN3 disease should have access to adapted, meaningful education, communication strategies, and leisure activities to maintain acquired skills for as long as possible to reduce frustration, isolation and boredom [5]. The ‘JNCL and Education Project’ was a multinational collaborative initiative involving parents, clinicians, educators, and social and health workers. Outcomes from that group included recommendations to implement proactive educational planning, with an emphasis on promoting adaptive skills to prolong function and independence as the disease progresses. An educational assessment tool was developed to identify individual strengths and to propose strategies for prolonging literacy and language skills. A key concept addressed by the group was participation in real-life activities, such as modified domestic duties, to maintain skills, enrichment, and QoL, particularly in the later stages of the disease [41]. Visual aid programmes, particularly Braille, when indicated, are recommended for inclusion in educational settings [114], as they increasingly affect the ability to read and write [147]. As an example of proactive learning, Braille should be introduced early in the disease course, perhaps while some vision remains, to optimise learning in advance of cognitive regression. A pragmatic approach is also necessary; some children may not be capable of acquiring Braille skills, but access to educational content should still be provided through other means.

The use of augmentative and alternative communication (AAC) strategies under the guidance and support of a qualified speech therapist should be considered and, where possible, adopted early. AAC is an important clinical intervention that may support patients in prolonging meaningful communication, interaction and engagement as speech and language skills regress. Speech therapy can also help develop facial and tongue muscles to support speech, language, feeding and swallowing. This therapy is conducted in the context of play and may incorporate music therapy. In a qualitative study of 13 children and adolescents with CLN3 and other forms of Batten disease, music therapy and singing were found to aid in the regulation and maintenance of speech and expressive language, movement control, enhance mood and promote social interaction, thus maintaining functional speech and communication for longer periods [143].

Eight statements were developed to support the social considerations statements for CLN3 disease, all of which reached a consensus.

**Table 5:** Social considerations, statements and consensus data

Statement	Responders	Evidence Level	Consensus %
Children should have the opportunities to access a wide variety of traditional educational tools, such as Braille, as well as new technologies, in order to both promote access to a full and meaningful educational curriculum and to enjoy a variety of leisure activities.	32	5	96
Adaptive communication support can be very valuable in helping patients with CLN3 disease maintain relationships and express their needs. When possible, speech and language experts should be involved as appropriate.	32	5	97
Supporting a patient’s physical health, functional status, and social interactions should remain a priority throughout the course of the disease. Accommodations may be needed to support mobility and communication, especially with regard to visual impairment, and strategies may vary between home, school, and community settings.	32	4	95
Augmentative and alternative communication (AAC) strategies should be considered and, where possible, adopted early. AAC is an important clinical intervention that may support patients in communication, interaction and engagement as speech and language skills regress.	31	5	89
Safe mobility and accessibility can be promoted through early adoption of supportive equipment and adaptive devices in consultation with a PT and/or OT, as appropriate. Aids may include white canes, orthoses, adaptive chairs and standing and walking equipment.	32	5	96
Caregiver strain is common and has a significant impact on families caring for loved ones with CLN3 disease. Multidisciplinary teams should have proactive strategies to screen for and address caregiver needs through support from social work, mental health resources, palliative medicine specialists and charitable resources.	32	4	95
Visual support is critical to maintaining function, and all measures should be employed to optimise the affected individual’s function.	32	4	93
Physical, occupational, speech and other supporting therapy interventions are recommended for patients to assist in maintaining the highest level of activity which may aid in quality of life (QoL).	32	3	96

### Ocular management (Table 6)

Rapid bilateral visual loss is an early clinical feature of CLN3 disease, although the ocular pathology in CLN3 disease remains poorly understood. Misdiagnoses for other ocular conditions, such as cone-rod dystrophy [148], cone dystrophy or Stargardt disease, are often made. Ophthalmologists should be aware of this rare condition, as early diagnosis facilitates access to social support and informed and prognostic genetic counselling [149].

Fundoscopy and OCT can be used to detect retinal abnormalities as part of the diagnostic workup for CLN3. Commonly observed changes include a “bull’s-eye” maculopathy, notable inner retinal loss of lamination, outer retinal thinning, peripheral pigmentary or atrophic changes, attenuation of retinal arterioles [57], and increased reflectivity [16]. Fundus examination may be normal in the early phases, contributing to diagnostic delays [57, 150]. Inflammation and vitreous reaction have been attributed to the rapid retinal degeneration seen in CLN3 disease; however, it remains unclear whether this is part of a broader immunological response [18].

CLN3 disease can be differentiated from the similarly presenting early‑onset Stargardt disease as visual acuity declines faster in CLN3 disease, and subtle neurological manifestations may be present. Dark‑adapted full‑field electroretinogram (ERG) responses are absent or electronegative in CLN3 disease but not in Stargardt disease. Subretinal flecks are a hallmark of Stargardt disease but are usually absent in CLN3 disease. OCT uncovers early loss of retinal lamination [151], which is not observed in Stargardt disease [7]. OCT also reveals optic disc pallor, with a specific macular striation pattern that can potentially discriminate CLN3 disease from other retinal dystrophies [152]. The advanced photoreceptor degeneration seen in CLN3 disease at diagnosis indicates that performing OCT early could reduce diagnostic delays [153]. Cataract formation and acute glaucoma can rarely appear in CLN3 disease [154].

Unlike most retinal dystrophies, CLN3 disease is also characterised by the death of cells in the ganglion cell layer. Perhaps insult in the dorsal lateral geniculate nucleus (LGNd) can lead to retrograde degeneration of the retina and ensuing atrophy [78]. Degenerate photoreceptors do not have autofluorescent pigments; thus, granule accumulation may not precede photoreceptor degeneration. Normal photoreceptor proteins in degenerate rods and cones suggest that they may regenerate [155]. Neuroinflammation may play a role in the neurodegeneration of the eye [18].

Retinal degradation correlates with increasing age and reduction in cortical GM, and regular ocular assessment is recommended by non-invasive SD-OCT when the individual is able to do so [156]. The recent development of the Hamburg CLN3 ophthalmic rating scale is a valuable tool for monitoring disease progression and evaluating the efficacy of future treatments [138].

Three statements were developed to support the ocular management statements of CLN3, all of which reached a consensus.

**Table 6:** Ocular management, statements and consensus data

Statement	Responders	Evidence Level	Consensus %
Rapid visual acuity loss, severe colour vision abnormalities and abnormal DA ERG responses are early clinical features of CLN3 disease and may assist in diagnosis but are not necessary.	32	4	75
Bull’s Eye Maculopathy, as well as retinitis pigmentosa (RP) features, can be observed in moderate to advanced stage. Early signs consist of foveal and parafoveal loss of the outer nuclear layer and inner nuclear layers.	31	4	75
Regular assessment of ocular disease severity using the Hamburg CLN3 ophthalmic rating scale may be a valuable research tool for the clinical evaluation of disease progression.	32	4	75

### Epilepsy / Seizures (Table 7)

As CLN3 disease progresses, seizure management is crucial to maintain QoL. Electroencephalography (EEG) confirms that seizure characteristics change over time [157]. Seizures remain infrequent, occurring less than once every three months. Generalised bilateral tonic‑clonic seizures are more common, followed by partial motor, complex partial, or absence seizures [158]. Myoclonic seizures are uncommon [157]. Most children only experience one seizure type. The frequency of seizures may or may not increase over time. Severity does not correlate with disease progression, although patients without seizures have a better QoL with less frequent hospitalisations [158].

Treating epilepsy in CLN3 disease with one or two anti‑seizure medications (ASMs) is usually successful. ASMs such as levetiracetam [42], sodium valproate, or lamotrigine as a monotherapy or in combination with clonazepam [159] reduce seizure frequency and severity [160]. One must keep in mind that ASMs come with potential side effects [161]. The healthcare team should be vigilant for valproate‑induced hyperammonemia/hyperammonemic encephalopathy in CLN3 patients. Hence, therapy must be tailored to the individual to maximise benefit and maintain QoL [162]. There is no literature on the newer ASMs in CLN3 disease.

Seizure episodes can be accompanied by a fearful expression that is similar to paroxysmal sympathetic hyperactivity (PSH). These symptoms are due to a disturbed somatosensory modulation leading to lower pain thresholds, degeneration in the anxiety/fear neural pathway, imbalance in the autonomic pathway, and dominance of the sympathetic neural system [163]. Continuous EEG monitoring can distinguish PSH from seizures, allowing for appropriate symptom management [164]. Regular seizure monitoring is recommended. PSH-like attacks may also appear in the absence of epileptic episodes and occur in response to separations, loud noises, or discomfort. Research into vagal nerve stimulation is warranted to better understand the sympathetic and parasympathetic neural balance in CLN3 disease [163]. Treatment is complex, but the frequency and intensity of the attacks can be reduced by minimising sensory provocation and using analgesic medication [44, 163].

Two statements were developed to support the management of seizures/epilepsy, both reaching a consensus.

**Table 7:** Epilepsy / seizures, statements and consensus data

Statement	Responders	Evidence Level	Consensus %
Epilepsy is a major feature of CLN3 disease, and epilepsy management by a neurologist or child neurologist is a key element in the care of CLN3 disease patients.	32	4	93
The most common seizure type in CLN3 disease is generalised tonic-clonic. In most patients, seizures can be reasonably well controlled with 1–2 appropriate anti-seizure medications and do not become progressively more treatment-resistant over time. Regular monitoring of seizure burden is important in order to adjust treatment for optimal control and to minimise adverse effects.	32	3	81

### Nutrition (Table 8)

As CLN3 disease progresses, swallowing difficulties arise, increasing the risk of food aspiration and pneumonia. As these difficulties progress, meeting the nutritional requirements and maintaining quality of life becomes more challenging. Therapeutic support should start as soon as swallowing difficulties occur. Guidelines have been developed to evaluate and treat nutritional complications in children with neurological impairment [165]. Caregivers should be educated in appropriate food consistencies and how to recognise the early signs of oro‑pharyngeal dysfunction. Gastrostomy feeding may maintain and support adequate nutrition and improve quality of life for patients and caregivers. This is not mandatory and should be discussed on an individual basis, according to the preferences of the family. Palliative medicine consultation may be helpful for families who are unsure about whether to pursue gastrostomy feeding, as this change can impact the patients’ and caregivers’ quality of life [165]. Oral secretions can be managed with pharmacological and non-pharmacological interventions [28]. Low‑dose botulinum toxin injection to the salivary glands may help minimise drooling but is seldom tolerated by children and their families.

Three statements were developed to support nutrition management in CLN3, all of which reached a consensus.

**Table 8:** Nutrition, statements and consensus data

Statement	Responders	Evidence Level	Consensus %
Adequate nutrition in CLN3 disease enhances the general well-being of patients, and growth parameters should be monitored alongside other aspects of care.	32	5	93
Many patients with CLN3 disease develop significant dysphagia as their disease progresses. Particularly once there is documented weight loss and/or episodes of aspiration pneumonia, medical teams should discuss the burdens and benefits of enteral tube feeding with families to see if this intervention is consistent with their expressed goals of care.	32	None	90
Tube feeding should be considered if one of the following is present: Increased risk of choking; Inability to meet nutritional requirements; Confirmed silent aspiration on video fluoroscopy; Repeated episodes of aspiration pneumonia confirmed by imaging.	32	None	90

### Respiratory health (Table 9)

In the latter stages of CLN3 disease, respiratory complications may arise and can quickly become life‑threatening. Respiratory symptoms are distressing and have a hugely negative impact on the burden of illness for the patient and their caregivers. Little data exists on the optimum management of respiratory symptoms in the paediatric palliative care setting. Good chest hygiene and management of secretions with mucolytics are important [166]. Once patients become non‑ambulatory, repositioning, postural drainage, physiotherapy, and, where necessary, ventilation support are important considerations [26].

Vaccinations against preventable respiratory diseases are recommended for the patient and their family in accordance with national guidelines [35].

Two statements were developed to support respiratory health in CLN3 disease, reaching a consensus.

**Table 9:** Respiratory health, statements and consensus data

Statement	Responders	Evidence Level	Consensus %
Respiratory complications can be a major threat to patients with CLN3 disease, particularly those that are non‑ambulatory and/or have difficulty protecting their airway. Interventions that help reduce the incidence and severity of respiratory infections include adequate positioning, chest physiotherapy, suctioning, and, in some cases, technology to support ventilation.	32	None	82
CLN3 children and their families/caregivers are recommended to receive all normal vaccinations.	32	4	95

### Sleep and rest (Table 10)

Sleep disorders are common in CLN3 disease, which markedly affects the quality of life for the entire family. Obtaining adequate sleep and rest is equally essential for the caregiver and the patient [167]. Sleep disturbances in CLN3 disease are multifactorial due to sleep onset delay, duration, night awakenings, and loss of vision, which impact the diurnal rhythm, parasomnias, and daytime sleepiness [168]. Sleep efficiency is also affected by lower percentages of rapid eye movement (REM) and non-REM sleep. These disturbances are likely due to psychological factors associated with CLN3 disease [169] and, in turn, exacerbate behavioural and cognitive impairments. Behavioural and environmental “sleep hygiene” strategies, with or without medications, may help treat sleep dysfunction [35]. The benefit of melatonin on sleep quality is controversial; a small study including three individuals with juvenile NCL (genetic diagnosis not reported) reported that melatonin use was not associated with changes to quantitative measurement via wrist actigraphy of sleep/wake activity patterns but that parents reported “slightly improved sleep quality” in their affected child [170]. Respite services and social groups provide emotional and mental health support for families and caregivers [95]. Such services are uncoordinated and underfunded in certain healthcare systems. Improving these services is necessary to lighten the burden on families and improve their quality of life [171]. Two statements were developed to support the recommendations for sleep and rest management, reaching a consensus.

**Table 10:** Sleep and rest, statements and consensus data

Statement	Responders	Evidence Level	Consensus %
Sleep disturbances are seen in patients with CLN3 disease and can negatively impact both the health of the patient as well as their families and caregivers. Strategies to screen for and manage insomnia and other night‑time symptoms should be part of ongoing care. For patients who remain ambulatory but have significant vision and/or cognitive impairment, include a discussion of overnight safety strategies aimed at reducing wandering and accidental harm.	32	4	96
Living with a rare disease is challenging for the whole family, and appropriate support and possibility for respite should be encouraged and offered as often as necessary to caregivers, siblings and family members.	32	4	97

### End-of-life care (Table 11)

The impact on the family and friends following a life-limiting diagnosis is profound. Palliative medicine in the paediatric and adult setting should be considered an integral component of the multidisciplinary care team from the time of diagnosis. Palliative care supports families to process information and adjust to the illness, helps to anticipate and optimally prepare management strategies for the next steps in the disease course, and supports QoL for the individual and their family throughout the entire course of the disease, not just in the terminal stage. Open and regular dialogue between the palliative care team and the families can help elicit the family’s perspective and priorities in a dynamic fashion. It can be particularly helpful when disease milestones occur, such as with the loss of vision, safe mobility, and the onset of dysphagia, as these changes often invoke the use of new medical technologies. These conversations should also include expectations for daily living and the family’s preferences for the use of language and metaphors [172]. As the individual’s condition deteriorates, the burden of care increases and less attention is available to other family members. Families report behavioural symptoms, vision loss, and communication impairments to be the most difficult to manage, with a significant impact on the quality of life for the whole family [167]. HCPs should be mindful that the transition into sharing care responsibilities with professionals can be difficult for some caregivers, who may feel a loss of control and a sense of ”letting go” of their children [172]. When an individual with CLN3 disease develops a high degree of health fragility, and there is a transition into comfort-focused care, hospice and home palliative care services should be offered, if they are available, allowing families QoL at the end of life. Many families prefer home‑based services, where the individual is comfortable and at peace in an environment they can control. Other families continue hospital‑based care through to the end of life in order to receive a high level of medical care even during the active dying process [35].

In the final stages, recurring hyperthermia unexplained by infection alternating with bouts of hypothermia, intermittent bradycardia, and cardiomyopathy may occur [64]. Symptom management in the advanced stages of CLN3 disease can present a significant challenge due to the complex nature of symptoms [1].

Paediatric palliative care continues to evolve globally, with recently developed standards highlighting the importance of interdisciplinary support, robust care models, and ongoing educational and advocacy efforts [173].

The goal of palliative care is to help people with a terminal illness live as well as possible for as long as possible while addressing suffering in all domains from the time of diagnosis through death and bereavement.

Three statements were developed to support the recommendation of end‑of‑life care, reaching a consensus.

**Table 11:** End-of-life care, statements and consensus data

Statement	Responders	Evidence Level	Consensus %
As CLN3 disease becomes advanced, patients may experience a high symptom burden and have medical complications that can be life-threatening, including aspiration events, refractory seizures, and cardiac arrhythmias. Support for patients during this time can be directed at minimising discomfort through treatment of pain and agitation, as well as helping to preserve function and interactions that the family identifies as meaningful. Support for family members and caregivers is critically important during this intense phase of illness, which can last months to years.	32	None	97
Palliative medicine offers supportive, multidisciplinary care for patients with CLN3 disease and their families from diagnosis to bereavement, helping to enhance the quality of life for patients for as long as possible in the context of progressive neurological illness. Some families engage with palliative medicine/supportive care teams as early as the time of diagnosis in order to help process and adjust to illness and to anticipate the next steps in management and care.	32	4	86
Palliative medicine specialists may help families plan specifically for end-of-life care if desired by a family, which may include support in the home or hospice if a family elects to focus on comfort care outside of a traditional hospital setting.	32	3	93

## Discussion

Clinical care recommendations for the management of CLN3 disease are intended to promote evidence‑based, comprehensive medical care for individuals and families affected by this rare disease. Early diagnosis of CLN3 disease enables more effective clinical management, guidance regarding the expected disease progression, informed genetic and psychological counselling, and family planning and support for patients and their families. Unless there is a positive family history, a CLN3 disease diagnosis is most commonly made following an ophthalmology referral for concerns about visual decline or progressive appearance of seizures, motor and cognitive decline [15].

This programme used a robust systematic approach to developing consensus‑based guidelines. The aim is to raise awareness of CLN3 disease, particularly in paediatric and ophthalmology settings, to accelerate early diagnosis rates and manage symptoms in line with the best evidence‑based standards of care. This methodology was implemented for the development of medical guidelines for other rare diseases, such as phenylketonuria [174], Maple Syrup Urine disease [175], and CLN2 disease [26]. An essential aspect of clinical recommendations is the inclusion of patient advocates to provide a voice for patients and their families.

Results in this report highlight the critical need for early diagnosis and greater awareness among paediatricians and ophthalmologists to recognise pathological symptoms indicating CLN3 disease and to differentiate from other similar conditions. CLN3 disease should be considered as a diagnosis for children who present with rapidly progressing bilateral vision loss, in adults with progressive neurological decline with a history of bilateral vision loss, and any individual with bilateral vision loss or retinitis pigmentosa (RP). The newly established Hamburg CLN3 ophthalmic rating scale may serve as an objective marker of ocular disease severity and progression and may be a valuable tool for evaluating novel therapeutic strategies for CLN3 disease [138]. Since our research was conducted, the International League Against Epilepsy have updated the classification of epileptic seizures, and these should be integrated into descriptions of CLN3 disease moving forward [176].

Patients should be referred to specialists or designated centres for diagnostic work‑up and ongoing management. Genetic screening can identify bi‑allelic pathogenic variants in the *CLN3* gene. Genetic counselling, early implementation of appropriate care plans, and facilitating access to ‘new’ treatments are essential for best clinical outcomes, and for holistic patient and family support [177].

For families without access to a designated centre, these guidelines may also be used to support management directed by the child’s primary physician. Established guidelines also serve as a means of family empowerment, helping parents and other caregivers advocate for care that aligns with published standards.

Currently, the main therapeutic options available for this disorder are limited to symptom management. In two case reports, bilateral pallidotomy with or without deep brain stimulation markedly improved dystonic storm. Neither case returned to pre‑pallidotomy levels, maintaining a reduction in abnormal movements [178]. Flupirtine has been shown to reduce apoptosis in CLN3‑deficient lymphocytes and neuronal cells in vitro [179]. There is some evidence that flupirtine leads to gender‑specific quantitative neuroanatomical and behavioural improvements in Cln3 ^Δ ex7–8^ mice [180, 181]. A parent‑perceived benefit was seen when children were given flupirtine, but no quantitative improvement was attributed to flupirtine. Further prospective research is required [182]. Dose escalation and safety trials have been considered for gene therapy in neurological diseases caused by *CLN3* gene mutation variants (NCT03770572), but are currently halted. No results have yet been published. Other disease‑modifying agents are currently underway. Information on clinical trials can be found at clinicaltrials.gov, and the EU Clinical Trials Information System (https://euclinicaltrials.eu/search-for-clinical-trials/?lang=en).

Recent and ongoing in-human interventional studies are continuing to advance the field. The whole spectrum of CLN3 disease is covered in these guidelines. Most currently known patients carry the 1‑kb deletion on at least one disease allele, present in childhood. The subject of gender differences did not meet a consensus in these guidelines, which may be due to conflicting publications [183].

The utility of telemedicine to monitor neurobehavioural assessments and to reduce the travel burden for families has been demonstrated in CLN3 disease and should be further explored [184, 185]. Telemedicine, especially during and following the COVID-19 pandemic, is in place in many countries and has proven useful for evaluating populations with impaired cognition and high care needs [117].

These guidelines do not include economic modelling. Local cost implications must be considered when implementing these guidelines. Scientifically proven innovative therapies, global collaborative translational medicine projects, and the DEM-CHILD collaborative database [186] will enable the continued evolution of research into the management and novel treatments for CLN3 disease.

### Strengths and limitations of the programme

The management of a person with CLN3 disease requires a coordinated multidisciplinary approach. As such, it is crucial that guidelines include a broad range of topics in the clinical and holistic management of this disease. Treatment advancements in rare diseases are hindered by the scarcity of high‑quality evidence for medical and treatment interventions. Thus, each consensus statement within these guidelines was assessed by the SC chairs using the Oxford Centre for Evidence‑Based Medicine (OCEBM) grading system. The response rate to each question was high, although not all HCPs responded to each question. Responders were from multiple disciplines and diverse cultural and geographical backgrounds. Not all questions were relevant to the expertise of each participant.

Multiple sponsors funded this programme, with the methods section describing how measures were implemented to ensure sponsors had no influence on the final statements.

The strength of these guidelines is due to the robust methodology in selecting the SC and chairs based on their prolific publication record and the EMT [30]. The multidisciplinary approach enabled the inclusion of experts globally, each bringing their knowledge of the condition and patient management, as well as local challenges concerning resources and capabilities. Input from two patient advocates was also included in the guideline development (Appendix 7). The comprehensive literature review conducted by both internal and external medical writers was repeated in light of the disruption to this programme caused by the COVID-19 pandemic. This ensured that the guidelines are based on current evidence‑based studies. The modified-Delphi voting process, designed to achieve consensus, ensured that each guideline statement represented the views of specialists from a range of disciplines. The guidelines aim to identify clinical management strategies, diagnostic methods, and holistic multidisciplinary care for use by HCPs, patients and their families. A risk of bias assessment was not undertaken, as it was not the purpose to gather information on efficacy outcomes. The methodology and transparency of the manuscript have been demonstrated via a review against the validated AGREE II instrument, where the guidelines gained a score of 6.4 (www.agreetrust.org).

### Future perspectives

These recommendations aim to reduce the diagnostic delay for patients with CLN3 disease and to optimise care for all patients, families and caregivers. The recommendations have been developed for international use as a basis that can be adapted to local policies. However, it is impossible to create audit guidance that is universally acceptable to all situations due to enormous variability in healthcare systems, geography, and economic and cultural differences. Therefore, an assessment of resource use was beyond the scope of these recommendations. The future objective is to boost medical education around rare paediatric neuropathic diseases [187]. Equally, an objective will be to identify clinicians, nurses, and other caregivers through meeting presentations, publications, online‑focused multi‑audience learning, and a website to summarise the recommendations outlined in these guidelines in an accessible format. These guidelines also constitute a link to other NCL resources and liaisons between HCPs and other stakeholders, such as education professionals, to ensure consistency, and aim to reduce global health inequity. The SC hopes that local groups will extract appropriate elements from these guidelines for implementation at their institutions and for their audit cycles. Audited information would be invaluable if it were available as feedback in regular reviews for updates to these guidelines. This was beyond the scope of this programme.

## Conclusions

This manuscript supplies robust evidence‑based and consensus‑driven guidelines for managing patients with CLN3 disease. These are available for use by all HCPs involved in the care of a patient with CLN3 disease, and for dissemination across the medical field, especially in medical training programmes. It is crucial to raise awareness of this destructive disease. These guidelines are applicable at this point in time. It will be necessary to implement updates as the knowledge evolves and evidence gaps are filled. The SC recommends that the guidelines be reviewed and updated within five years or sooner if significant developments lead to treatments or changes in medical practice, which may further the development of the criteria for monitoring and auditing purposes. This update may be in the form of a renewed systematic literature review in the first instance. If pertinent data emerges, amendments to these guidelines will be published.

This guideline programme addresses unmet clinical needs for patients with CLN3 disease and their families. The manuscript is designed to be readily accessible online as one element to accompany other available resources, including plain language summaries and information on patient support groups. It is intended that the robust methodology employed can be transferred to developing guidelines for other rare diseases.

## Supplementary Information

Below is the link to the electronic supplementary material.

Supplementary Material 1

Supplementary Material 2

Supplementary Material 3

Supplementary Material 4

Supplementary Material 5

Supplementary Material 6

Supplementary Material 7

## Data Availability

The datasets used and/or analysed during the current study are available from the corresponding author upon reasonable request.

## Abbreviations

6MWT: 6-Minute Walk Test
AAC: Augmentative and Alternative Communication
ASM: Anti-Seizure Medication
AGREE II: Appraisal of Guidelines, Research and Evaluation (II)
CLN3: Ceroid Lipofuscinosis
EMT: Expert Mapping Tool
BDSRA: Batten Disease Support and Research Association
BDFA: Batten Disease Family Association
JNCL: Juvenile Neuronal Ceroid Lipofuscinosis
LAMP-1: Lysosomal-Associated Membrane Protein-1
MDT: Multidisciplinary Team
NCL: Neuronal Ceroid Lipofuscinosis
obsRO: Observer-Reported Outcome Measures
OCEBM: Oxford Centre for Evidence-Based Medicine
OCT: Optical Coherence Tomography
P.I.C.O: Problem/Patient/Population, Intervention/Indicator, Comparison, and Outcome
PPC: Paediatric Palliative Care
PRISMA: Preferred Reporting Items for Systematic Reviews and Meta-Analyses
PSH: Paroxysmal Sympathetic Hyperactivity
QoL: Quality of Life
RP: Retinitis Pigmentosa
SC: Steering Committee
UBDRS: Unified Batten Disease Rating Scale
