# Supplementary material for: Recommendations for the diagnosis and management of cln3 disease (batten disease) using the Delphi consensus methodology
Source: Orphanet J Rare Dis. 2026 Mar 10;21:155. doi: 10.1186/s13023-026-04298-2 (PMC13088526; doi:10.1186/s13023-026-04298-2)
Supplement: Supplementary file 1 — Supplementary Material 1 [file 13023_2026_4298_MOESM1_ESM.docx]

**Appendices**

**Appendix 1**

Table of Scientific Meetings in the five years preceding project starting (2021)

| Events Included | Event Frequency | Years Included | | | | |
| --- | --- | --- | --- | --- | --- | --- |
| SSIEM | Annually | **2019** | **2018** | **2017** | ~~2016~~ | **2015** |
| CNS | Annually | **2019** | **2018** | **2017** | **2016** | **2015** |
| WORLDS | Annually | **2020** | **2019** | **2018** | **2017** | **2016** |
| International Conference on Neuronal Ceroid Lipofuscinosis | Bi-Annual | **2016** | **2018** |  |  |  |
| ESGLD Workshop | Bi-Annual | **2015** | **2017** |  |  |  |
| ICIEM | 1 every 4 Years with SSIEM |  |  |  |  |  |
| American Epilepsy Society | Annually | **2016** | **2017** |  |  | **2020** |
| ACMG | Annually | None Available | | | | |
| EAPS | Bi-Annual | **2020** | **2018** | **2016** |  |  |
| Lysosomal Disease Conference | Bi-Annual | **2015** | **2017** | **2019** |  |  |
| EPNS Congress | Bi-Annual | **2017** | **2019** |  |  |  |
| EPNS Research Meeting | Bi-Annual | **2016** | **2018** |  |  |  |
| ASHG | Annually | **2020** |  |  |  |  |
