# Supplementary material for: Recommendations for the diagnosis and management of cln3 disease (batten disease) using the Delphi consensus methodology
Source: Orphanet J Rare Dis. 2026 Mar 10;21:155. doi: 10.1186/s13023-026-04298-2 (PMC13088526; doi:10.1186/s13023-026-04298-2)
Supplement: Supplementary file 2 — Supplementary Material 2 [file 13023_2026_4298_MOESM2_ESM.docx]

**Appendix 2**

**Search Terms for the Systematic Literature Review.**

**PubMed**

Medical Subject Headings (MeSH): ceroid lipofuscinosis-neuronal, ceroid lipofuscinosis-neuronal juvenile type, and Batten disease.

Free text:

((CLN3[Title/Abstract]) AND (diagnos* OR clinical OR aware* OR manage*); (JNCL[Title/Abstract]) AND (diagnos* OR clinical OR aware* or manage*); ('juvenile neuronal ceroid lipofuscinosis'[Title/Abstract] OR JNCL[Title/Abstract]) AND (diagnos* OR clinical OR aware* OR manage*); ('neuronal ceroid lipofuscinosis'[Title/Abstract] AND 3 [Title/Abstract]) AND (diagnos* OR clinical OR aware* OR manage*); ('juvenile batten disease'[Title/Abstract]) AND (diagnos* OR clinical OR aware* OR manage*); vogt-spielmeyer; spielmeyer AND sjogren.

**EMBASE**

Emtree: ceroid lipofuscinosis-neuronal, ceroid lipofuscinosis-neuronal juvenile type, and Batten disease.

Free text:

((CLN3[Title/Abstract]) AND (diagnos* OR clinical OR aware* OR manage*); (JNCL[Title/Abstract]) AND (diagnos* OR clinical OR aware* or manage*); ('juvenile neuronal ceroid lipofuscinosis'[Title/Abstract] OR JNCL[Title/Abstract]) AND (diagnos* OR clinical OR aware* OR manage*); ('neuronal ceroid lipofuscinosis'[Title/Abstract] AND 3 [Title/Abstract]) AND (diagnos* OR clinical OR aware* OR manage*); ('juvenile batten disease'[Title/Abstract]) AND (diagnos* OR clinical OR aware* OR manage*); vogt-spielmeyer; spielmeyer AND sjogren.
