# Supplementary material for: Recommendations for the diagnosis and management of cln3 disease (batten disease) using the Delphi consensus methodology
Source: Orphanet J Rare Dis. 2026 Mar 10;21:155. doi: 10.1186/s13023-026-04298-2 (PMC13088526; doi:10.1186/s13023-026-04298-2)
Supplement: Supplementary file 4 — Supplementary Material 4 [file 13023_2026_4298_MOESM4_ESM.docx]

**Appendix 4:**

**PRISMA Flow Diagram: Internal Medical Writer**

**Identification of studies via databases and registers**

Records removed *before screening*:

Duplicate records removed (n = 700)

Records marked as ineligible by automation tools (n = 0)

Records removed for other reasons (n = 0)

Records identified from*:

EMBASE (n = 3658)

Minus duplicates (n = 2394)

PubMed (n = 2348)

Minus duplicates (n = 1442)

**Identification**

Records screened

(n = 2170)

Records excluded**

Mendeley (n = 700)

Reports sought for retrieval

(n = 1470)

Reports not retrieved

(n = 0)

**Screening**

Reports excluded:

Animal studies (n =324)

In Vitro (n =243)

Narrative Reviews (n = 109)

Other: Conference proceedings, Not CLN3, comments/errors/retired (n=551)

Reports assessed for eligibility

(n = 1470)

Studies included in review

(n = 243)

**Included**

*Consider, if feasible to do so, reporting the number of records identified from each database or register searched (rather than the total number across all databases/registers).

**If automation tools were used, indicate how many records were excluded by a human and how many were excluded by automation tools.

*From:*  Page MJ, McKenzie JE, Bossuyt PM, Boutron I, Hoffmann TC, Mulrow CD, et al. The PRISMA 2020 statement: an updated guideline for reporting systematic reviews. BMJ 2021;372:n71. doi: 10.1136/bmj.n71

For more information, visit: <http://www.prisma-statement.org/>
